# Deep learning-radiomics assessment of intervertebral disc and paraspinal muscle heterogeneity for predicting postoperative recurrent lumbar disc herniation

**DOI:** 10.3389/frai.2026.1757269

**Published:** 2026-02-04

**Authors:** Guangdong Zhang, Ziqian Zhu, Haiyan Zheng, Xindong Chang, Fanyi Zeng, Jianwei Cui, Ming Tang, Shiwu Yin

**Affiliations:** 1 Department of Interventional Vascular Medicine, The Second People’s Hospital of Hefei, Hefei Hospital Affiliated to Anhui Medical University, Hefei, Anhui, China; 2 The Fifth Clinical College of Medicine, Anhui Medical University, Hefei, Anhui, China; 3 Department of Neurology (Sleep Disorders), The Affiliated Chaohu Hospital of Anhui Medical University, Hefei, Anhui, China; 4 Department of Interventional Vascular Medicine, Hospital of Anhui Corps, Chinese People’s Armed Police Force, Hefei, Anhui, China; 5 Graduate School, Zunyi Medical University, Zunyi, Guizhou, China

**Keywords:** deep learning, intervertebral disc, lumbar disc herniation, paraspinal muscle, postoperative recurrent lumbar disc herniation

## Abstract

**Objective:**

Although imaging and paraspinal muscle parameters are linked to postoperative recurrent lumbar disc herniation (PRLDH), micro-level texture characteristics and their interactions remain underexplored. This study applied deep learning (DL)-radiomics to quantify the microstructural heterogeneity of responsible intervertebral discs and paraspinal muscles (L3-S1), and assessed a combined disc-muscle model for predicting PRLDH.

**Method:**

Clinical and imaging data from 170 lumbar disc herniation (LDH) patients undergoing percutaneous transforaminal endoscopic surgery (Jan 2022-Dec 2024) were retrospectively analyzed. DL and radiomics features were extracted from intervertebral discs and paraspinal muscles. Feature selection via mutual information was followed by construction of a DL-radiomics Radscore model. Internal validation used leave-one-out, 10-fold cross-validation, and bootstrapping. Pfirrmann grading performance was compared with the disc Radscore, and potential disc-muscle interactions were explored using optimal cutoffs.

**Results:**

Among 170 patients, 39 had postoperative recurrence. Disc Radscore included 2 DL and 3 radiomics features, while muscle Radscore comprised 2 DL and 5 radiomics features. The disc Radscore demonstrated good predictive ability (AUC 0.857, 95% CI 0.797–0.918) across validation methods (AUC 0.846–0.857). Muscle Radscore showed moderate performance (AUC 0.718, 95% CI 0.627–0.809). Pfirrmann grade poorly predicted recurrence (AUC 0.506, 95% CI 0.412–0.600). Combined disc-muscle analysis was less stable than disc Radscore alone.

**Conclusion:**

DL-radiomics-derived intervertebral disc Radscore robustly predicts PRLDH. While combined disc-muscle assessment is less consistent, their interactions may inform postoperative risk stratification and management in LDH patients.

## 1 Introduction

Despite the effectiveness of surgical intervention, postoperative recurrent lumbar disc herniation (PRLDH) continues to pose a significant challenge for patients with lumbar disc herniation (LDH) (Nakamura and Yoshihara, 2017). Identifying patients at high risk is essential for tailoring postoperative strategies. Although Pfirrmann grading remains the reference standard for assessing disc degeneration, it reflects only macroscopic structural changes and is influenced by subjective interpretation (Rim, 2016). Moreover, evidence regarding the relationship between Pfirrmann grade and PRLDH is inconsistent (Li et al., 2023; Tang et al., 2022). Spinal stability relies on the integrated function of discs, paraspinal muscles, and neural elements (Panjabi, 1992). While previous research has established a link between muscle degeneration and PRLDH, most studies have been limited to morphological or macroscopic texture analysis at a single level, such as L4-L5, overlooking the biomechanical role of the entire lumbar musculature and the value of microstructural texture features (Tang et al., 2024; Tekin et al., 2025; Kong et al., 2020; Sun et al., 2025).

Given the strength of deep learning (DL)-radiomics in quantifying subtle tissue heterogeneity (Zheng et al., 2022), this study set out to construct models for both the responsible intervertebral disc and the paraspinal muscles spanning L3 to S1. We hypothesized that quantitative DL-radiomic features extracted from the intervertebral disc and paraspinal muscles could capture microstructural alterations associated with PRLDH, thereby providing superior predictive performance compared with the conventional Pfirrmann grading system. Accordingly, our objectives were threefold: (i) to evaluate how well the intervertebral disc Radscore predicts PRLDH and compare it with Pfirrmann grading; (ii) to assess the predictive performance of the paraspinal muscle Radscore; (iii) to examine whether combining disc and muscle features could offer meaningful insights for postoperative risk stratification. The study followed the reporting structure recommended by the Imaging Biomarker Standardization Initiative (IBSI) (Supplementary material S1).

## 2 Methods

### 2.1 Patients

This study retrospectively included patients with LDH who were treated at our hospital’ s Interventional Pain Department from January 2022 to December 2024. Inclusion criteria were as follows: (1) diagnosis of LDH according to established criteria (Basic, Research, Professional Committee of Spine Transformation Society, and Chinese Association of Rehabilitation Medicine Spinal Cord, 2022); (2) symptom duration ≥3 months with failure of conservative treatment; and (3) preoperative confirmation by MRI with available L3-S1 CT imaging, treatment with percutaneous transforaminal endoscopic surgery, and no prior surgery at the affected level. Exclusion criteria included: (1) previous lumbar spine surgery; (2) spinal tumors, tuberculosis, deformities, or fractures affecting spinal structure; (3) long-term postoperative medication use potentially influencing paraspinal muscles; (4) inability to identify the responsible disc; (5) incomplete clinical data; and (6) severe cardiovascular, cerebrovascular, or other congenital diseases. The study complied with the Declaration of Helsinki and was approved by our institutional ethics committee (2023-Keyan-062). Recurrence was defined as the reappearance of neurologic symptoms on the same side and segment, confirmed by imaging, occurring at least 6 months postoperatively (Kim et al., 2019). Follow-up lasted 6 months and was conducted through outpatient visits, review of electronic medical records, and telephone contact. Pain severity was measured using the Visual Analog Scale (VAS) preoperatively and on postoperative day 3, with higher scores indicating greater pain (Tascioglu and Sahin, 2022).

### 2.2 Clinical characteristics

General variables included gender, age, disease duration, occupation, smoking status, diabetes, and hypertension. Perioperative variables were preoperative and postoperative VAS scores. Imaging features comprised Pfirrmann grade and Modic changes.

Pfirrmann grading on T2-weighted sagittal images was defined as: Grade I: normal disc structure and height, bright signal; Grade II: abnormal disc structure with normal height, bright signal, and clear nucleus-annulus boundary; Grade III: abnormal structure, normal or slightly reduced height, intermediate signal, unclear boundary; Grade IV: abnormal structure, normal or moderately reduced height, dark signal, and absent boundary; Grade V: collapsed disc with abnormal structure and no visible nucleus-annulus distinction.

Modic changes were classified on T1- and T2-weighted sagittal images. Normal: equal T1WI and T2WI signal; Type I: low T1WI, high T2WI; Type II: high T1WI, high or equal T2WI; Type III: low signal on both T1WI and T2WI.

### 2.3 Image acquisition and segmentation methods

All patients underwent preoperative MRI (3.0 T, Siemens, Germany) and CT (64-slice, Siemens, Germany) scans in the supine position, with T2-weighted sagittal images acquired. The images were imported into 3D Slicer 5.8.1. Two interventional physicians, one junior (GZ) and one senior (SY), independently outlined the ROIs of the responsible intervertebral disc. For the L3-S1 paraspinal muscles-including the multifidus, erector spinae, and psoas major-the ROIs were drawn using a semi-automatic approach. Any disagreements were settled through discussion and consensus.

### 2.4 Deep learning methods and traditional omics features

UCTransNet is a semantic segmentation network based on the U-Net architecture, which incorporates the Channel-wise Cross Attention Transformer (CCT) to replace conventional skip connections (Wang et al., 2022). By leveraging the Channel-wise Cross-fusion Attention (CCA) mechanism within the CCT, the network effectively bridges the semantic gap and improves feature representation. The core formulation of the CCA module is as follows:


$$E_{\text{out}}=\text{Softmax}(\frac{(EW_{Q}){\left(EW_{K}\right)}^{T}}{\sqrt{d}})(EW_{V})+E$$

$E_{\text{out}}$ denotes the output feature map, and E represents the input feature map. $W_{Q}$ , $W_{K}$ , and $W_{V}$ are learnable weight matrices. $(EW_{Q}){\left(EW_{K}\right)}^{T}$ represents the attention mechanism, while $EW_{V}$ corresponds to the weighted aggregation. E serves as a residual connection. The proposed segmentation architecture consists of an encoding stage with four down-sampling layers and a decoding stage with four up-sampling layers. The overall formulation is expressed as follows:


$$E_{i}\in {\mathbb{R}}^{C_{i}\times H_{i}\times W_{i}}$$

Here, $C_{i}$ denotes the number of channels, and $H_{i}$ and $W_{i}$ represent the spatial dimensions of the feature map $E_{i}$ at the *i*-th layer. Each down-sampling and up-sampling layer comprises two grouped convolutional blocks. Each grouped convolution consists of a 3 × 3 kernel convolution, followed by a batch normalization layer and a ReLU activation. Network parameters were optimized using the Adam optimizer with an initial learning rate of 0.0001 and a weight decay of 1e-4 to prevent overfitting. The batch size was set to 32, and the training process was conducted for 50 epochs. The model achieving the best performance on the validation set was retained for feature extraction. The network was trained using a combined loss function of binary cross-entropy and Dice loss.

DL features were extracted from ROIs for predicting PRLDH. The DL feature extraction process was performed as follows: (i) ROI selection: Rectangular ROIs covering the tissue were obtained for DL analysis. All ROIs were resized to a uniform dimension of 224 × 224 pixels and used as input images. (ii) Image normalization: Input images were normalized using min-max scaling according to the following formula:


$$X^{*}=(\frac{X-X_{m}}{X_{M}-X_{m}})$$

**Figure 1:**
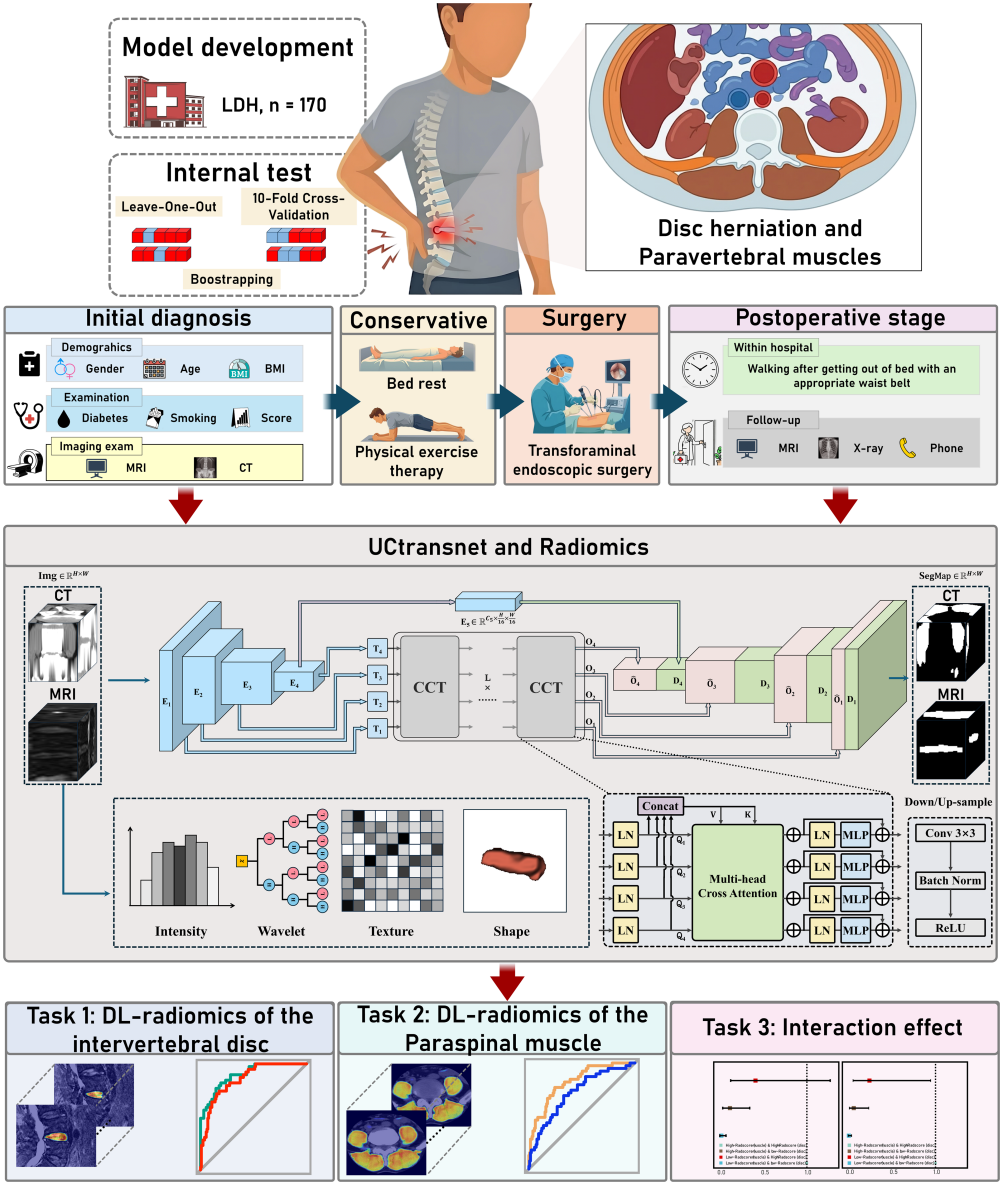
Workflow of radiomics and deep-learning (DL) feature extraction, image segmentation, DL-radiomics model building, feature selection, and result analysis. CCT, Channel-wise Cross-Attention Transformer; LDH, lumbar disc herniation.

Where X represents the original pixel intensity, $X_{M}\;\text{and}\;X_{m}$ are the maximum and minimum pixel values in the original image, respectively, and $X^{*}$ denotes the normalized pixel intensity. (iii) Representative feature extraction: The normalized 2D images were input into the DL network, and feature maps were extracted from the fourth downsampling activation layer of UCTransNet. Global average pooling was applied to obtain a 1 × 512-dimensional semantic segmentation feature for each 2D image. The DL feature extraction process comprised two modules: a DL feature extraction module and a deep feature selection module. The workflow is illustrated in Figure 1. First, the network was trained on the segmentation dataset to capture lesion-specific features. During testing, 2D images were input into the trained DL network, and feature maps were extracted from the fourth downsampling activation layer of UCTransNet. Global average pooling was then performed to generate DL features. Second, features extracted from the segmentation dataset were used to construct a feature library for adaptive similarity evaluation. Finally, an unsupervised clustering algorithm was applied to divide features into two clusters, and the similarity between the clusters and the feature library was evaluated to select the most informative feature combinations. A total of 512 DL features were extracted from each patient for each parameter map. UCTransNet was implemented using PyTorch 2.3.1 + CUDA 11.8 and executed on an NVIDIA RTX 2080 Ti GPU.

Radiomics features were extracted using PyRadiomics, encompassing shape, first-order statistics, gray-level co-occurrence matrix (GLCM), and gray-level run length matrix (GLRLM) features. Image processing included original images, wavelet decomposition, Laplacian of Gaussian (LoG) filtering, square, square root, logarithm, and exponential transformations, yielding a total of 1,223 features.

### 2.5 Statistical analysis

Statistical analyses were performed using R Studio (v4.2.3) and Python (v3.9.13). Continuous variables were expressed as mean ± standard deviation (*x̄* ± s) and compared using independent *t*-tests. Categorical variables were presented as counts and percentages (n %) and analyzed using the chi-square test.

Radiomics and DL features were extracted through a stepwise procedure. First, features from ROIs outlined by two radiologists were assessed with the intraclass correlation coefficient (ICC), retaining those with ICC > 0.75. Next, the Maximum Relevance Minimum Redundancy (MRMR) algorithm combined with a random forest classifier (5-fold cross-validation) was used to remove redundant features. Features with high inter-feature correlation (Pearson > 0.9) or weak association with outcomes (<0.3) were further excluded. Finally, LASSO and SVM-RFE with 10-fold cross-validation were applied to narrow the feature set. The final DL Radiomics features were used to calculate a Radscore, representing the DL Radiomics model (Figure 1).

The discriminative performance was assessed using the Area Under the Curve (AUC). To evaluate the added value of clinical factors, we constructed “Adjusted Models” using multivariate logistic regression, incorporating the Radscores along with clinical covariates. Model calibration (the agreement between predicted probabilities and observed frequencies) was assessed using calibration curves and the Hosmer-Lemeshow goodness-of-fit test. To ensure robustness and avoid overfitting, the performance of both Unadjusted and Adjusted models (including the multivariate fitting process) was validated using leave-one-out, 10-fold cross-validation, and bootstrap validation (1,000 resamples). Cutoff values for the Radscores were determined using the Youden index. All models were adjusted for age, sex, BMI, diabetes, hypertension, smoking history, pre- and postoperative VAS scores, disease duration, occupation, Pfirrmann grade, Modic changes, herniation type, and herniation segment. Differences were considered statistically significant at *p* < 0.05.

## 3 Results

### 3.1 General results

A total of 170 patients were enrolled, with ages ranging from 21 to 88 years (mean 58.44 ± 14.38 years). Among them, 39 patients experienced postoperative recurrence, with ages between 30 and 87 years (mean 59.00 ± 13.64 years). Significant differences were observed between the PRLDH and non-PRLDH groups in disease duration, Intervertebral Disc Radscore, Paraspinal Muscle Radscore, and combined Intervertebral Disc and Paraspinal Muscle Radscore (*p* < 0.05). The detailed comparison of patient characteristics is summarized in Table 1.

**Table 1:** Characteristics of PRLDH and non-PRLDH.

Variables	Total (*n* = 170)	Non-PRLDH (*n* = 131)	PRLDH (*n* = 39)	*t*/χ^2^/−	*P*
Age (years)	58.44 ± 14.38	58.27 ± 14.64	59.00 ± 13.64	−0.28	0.781
Gender (%)				1.69	0.194
Female	72 (42.35)	59 (45.04)	13 (33.33)		
Male	98 (57.65)	72 (54.96)	26 (66.67)		
BMI (kg/m^2^)	24.65 ± 3.40	24.44 ± 3.46	25.37 ± 3.12	−1.51	0.134
Smoking (%)				1.01	0.315
No	124 (72.94)	98 (74.81)	26 (66.67)		
Yes	46 (27.06)	33 (25.19)	13 (33.33)		
Disease duration (years)	4.06 ± 6.08	3.46 ± 5.41	6.05 ± 7.66	−2.36	0.019
Diabets (%)				2.41	0.121
No	133 (78.24)	106 (80.92)	27 (69.23)		
Yes	37 (21.76)	25 (19.08)	12 (30.77)		
Hypertension (%)				0.25	0.620
No	106 (62.35)	83 (63.36)	23 (58.97)		
Yes	64 (37.65)	48 (36.64)	16 (41.03)		
Preoperative VAS score (%)				–	0.542
3	1 (0.59)	1 (0.76)	0 (0.00)		
4	8 (4.71)	5 (3.82)	3 (7.69)		
5	79 (46.47)	62 (47.33)	17 (43.59)		
6	68 (40.00)	53 (40.46)	15 (38.46)		
7	10 (5.88)	6 (4.58)	4 (10.26)		
8	4 (2.35)	4 (3.05)	0 (0.00)		
Postoperative VAS score (%)				0.01	0.908
2	134 (78.82)	103 (78.63)	31 (79.49)		
3	36 (21.18)	28 (21.37)	8 (20.51)		
Occupation (%)				–	0.899
Office worker	63 (37.06)	47 (35.88)	16 (41.03)		
Laborer	15 (8.82)	11 (8.40)	4 (10.26)		
Farmer	72 (42.35)	57 (43.51)	15 (38.46)		
Self-employed households	20 (11.76)	16 (12.21)	4 (10.26)		
Pfirrmann grade (%)				0.02	0.992
Grade III	64 (37.65)	49 (37.40)	15 (38.46)		
Grade IV	88 (51.76)	68 (51.91)	20 (51.28)		
Grade V	18 (10.59)	14 (10.69)	4 (10.26)		
Modic change (%)				–	0.561
Normal	45 (26.47)	36 (27.48)	9 (23.08)		
Type I	1 (0.59)	1 (0.76)	0 (0.00)		
Type II	107 (62.94)	83 (63.36)	24 (61.54)		
Type III	17 (10.00)	11 (8.40)	6 (15.38)		
Herniation type (%)				2.27	0.321
Bulge	26 (15.29)	19 (14.50)	7 (17.95)		
Herniation	130 (76.47)	99 (75.57)	31 (79.49)		
Prolapse	14 (8.24)	13 (9.92)	1 (2.56)		
Herniation segments (%)				–	0.187
L3/5	6 (3.53)	6 (4.58)	0 (0.00)		
L4/5	107 (62.94)	85 (64.89)	22 (56.41)		
L5/S1	57 (33.53)	40 (30.53)	17 (43.59)		
Intervertebral disc Radscore	−1.41 ± 1.90	−1.95 ± 1.64	0.40 ± 1.54	−7.94	<0.001
Paraspinal muscle Radscore	−1.23 ± 1.16	−1.44 ± 1.07	−0.54 ± 1.19	−4.46	<0.001
Intervertebral disc and Paraspinal muscle Radscore (%)				51.95	<0.001
High-Radscore (muscle) and High-Radscore (disc)	31 (18.24)	11 (8.40)	20 (51.28)		
Low-Radscore (muscle) and High-Radscore (disc)	21 (12.35)	12 (9.16)	9 (23.08)		
High-Radscore (muscle) and Low-Radscore (disc)	39 (22.94)	32 (24.43)	7 (17.95)		
Low-Radscore (muscle) and Low-Radscore (disc)	79 (46.47)	76 (58.02)	3 (7.69)		

BMI, Body Mass Index; *t*, *t*-test; χ^2^, Chi-square test; −, Fisher exact.

### 3.2 Feature selection results of deep learning-radiomics

After ICC-based screening, 813 PyRadiomics features for the disc and 921 for the paraspinal muscles, as well as 201 DL features for the disc and 263 for the muscles, were retained. The MRMR algorithm combined with a random forest classifier (5-fold cross-validation) further reduced the sets to 12 and 8 PyRadiomics features and 7 and 3 DL features. Features with high inter-feature correlation (Pearson > 0.9) or low association with outcomes (<0.3) were excluded, leaving 11 and 8 PyRadiomics features and 6 and 3 DL features. Finally, LASSO and SVM-RFE (10-fold cross-validation) narrowed the feature sets to 5 and 3 PyRadiomics features and 2 and 2 DL features. The Radscore calculation formula is detailed in Supplementary material S2.

### 3.3 Evaluation of the predictive performance of the responsible intervertebral disc and paraspinal muscles

**Figure 2:**
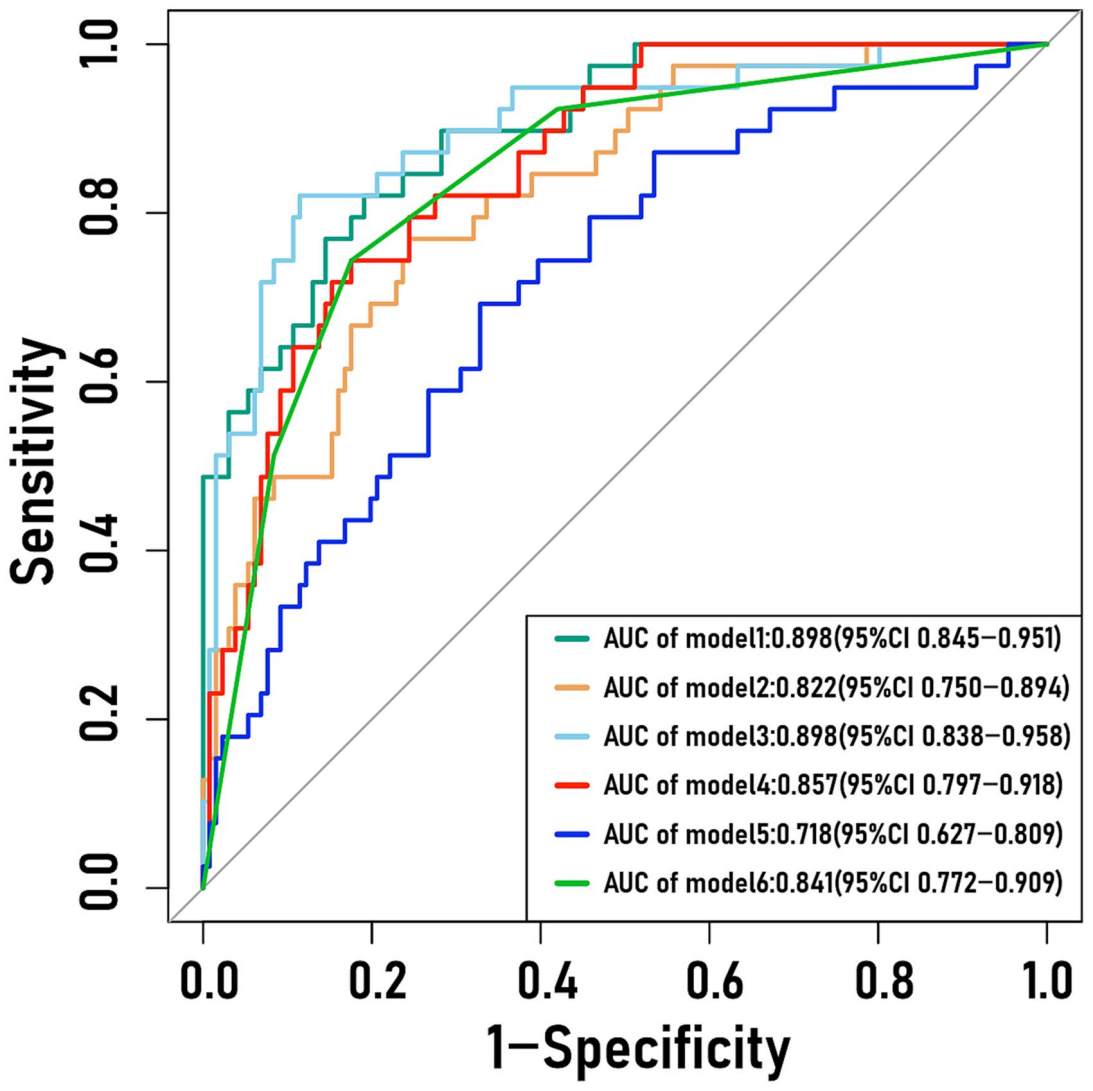
Unadjusted and adjusted ROC curves of the intervertebral disc Radscore, paraspinal muscle Radscore, and the combined model. Model 1: Intervertebral disc Radscore (adjusted); Model 2: Paraspinal muscle Radscore (adjusted); Model 3: Intervertebral disc and paraspinal muscle (adjusted); Model 4: Intervertebral disc Radscore (unadjusted); Model 5: Paraspinal muscle Radscore (unadjusted); Model 6: Intervertebral disc and paraspinal muscle (unadjusted). Adjusted: age, gender, BMI, diabetes, hypertension, smoking, pre- and post-operative VAS scores, disease duration, occupation, Pfirrmann grade, Modic changes, herniation type, and herniation segment.

As shown in Tables 2, 3 and Figure 2, the Intervertebral Disc Radscore achieved an AUC of 0.857 (95% CI 0.797–0.918), with an accuracy of 0.806, sensitivity 0.744, and specificity 0.824. Internal validation confirmed robust performance, with AUCs of 0.846 (leave-one-out), 0.847 (10-fold cross-validation), and 0.857 (bootstrap). After adjusted, the AUC increased to 0.898, with accuracy 0.812, sensitivity 0.821, and specificity 0.809. By contrast, the Pfirrmann grade performed poorly, with an AUC of 0.506, accuracy 0.571, sensitivity 0.385, and specificity 0.626.

**Table 2:** Model prediction performance.

Model	AUC (95%CI)	Accuracy (95%CI)	Sensitivity (95%CI)	Specificity (95%CI)	PPV (95%CI)	NPV (95%CI)
Pfirrmann grade						
Unadjusted	0.506 (0.412, 0.600)	0.571 (0.493, 0.646)	0.385 (0.234, 0.554)	0.626 (0.537, 0.709)	0.234 (0.138, 0.357)	0.774 (0.682, 0.849)
Adjusted	0.719 (0.628, 0.811)	0.700 (0.625, 0.768)	0.641 (0.472, 0.788)	0.718 (0.632, 0.793)	0.403 (0.281, 0.536)	0.870 (0.792, 0.927)
Intervertebral disc Radscore						
Unadjusted	0.857 (0.797, 0.918)	0.806 (0.738, 0.863)	0.744 (0.579, 0.870)	0.824 (0.748, 0.885)	0.558 (0.413, 0.695)	0.915 (0.850, 0.959)
Adjusted	0.898 (0.845, 0.951)	0.812 (0.745, 0.868)	0.821 (0.665, 0.925)	0.809 (0.731, 0.873)	0.561 (0.424, 0.693)	0.938 (0.877, 0.975)
Paraspinal muscle Radscore						
Unadjusted	0.718 (0.627, 0.809)	0.677 (0.601, 0.746)	0.692 (0.524, 0.830)	0.672 (0.584, 0.751)	0.386 (0.272, 0.510)	0.880 (0.800, 0.936)
Adjusted	0.822 (0.750, 0.894)	0.759 (0.687, 0.821)	0.769 (0.607, 0.889)	0.756 (0.673, 0.827)	0.484 (0.355, 0.614)	0.917 (0.848, 0.961)
Intervertebral disc and Paraspinal muscle						
Unadjusted	0.841 (0.772, 0.909)	0.806 (0.738, 0.863)	0.744 (0.579, 0.870)	0.824 (0.748, 0.885)	0.558 (0.413, 0.695)	0.915 (0.850, 0.959)
Adjusted	0.898 (0.838, 0.958)	0.871 (0.811, 0.917)	0.821 (0.665, 0.925)	0.885 (0.818, 0.934)	0.681 (0.529, 0.809)	0.943 (0.886, 0.977)

Unadjusted: raw model; Adjusted: adjusted for age, gender, BMI, diabetes, hypertension, smoking status, pre- and postoperative VAS scores, disease duration, occupation, Pfirrmann grade, Modic changes, herniation type, and herniation segment.

**Table 3:** Internal validation of the model.

Model	Leave-one-out cross-validation		10-fold cross-validation		Boostrapping	
	AUC	95%CI	AUC	95%CI	AUC	95%CI
Intervertebral disc Radscore						
Unadjusted	0.846	0.783, 0.910	0.847	0.783, 0.911	0.857	0.766, 0.932
Adjusted	0.824	0.750, 0.898	0.825	0.750, 0.900	0.801	0.677, 0.908
Paraspinal muscle Radscore						
Unadjusted	0.701	0.606, 0.795	0.699	0.602, 0.795	0.719	0.590, 0.834
Adjusted	0.702	0.610, 0.793	0.696	0.604, 0.787	0.689	0.561, 0.821
Intervertebral disc and Paraspinal muscle						
Unadjusted	0.764	0.672, 0.856	0.791	0.706, 0.876	0.833	0.729, 0.928
Adjusted	0.811	0.729, 0.894	0.824	0.746, 0.901	0.784	0.654, 0.904

Unadjusted: raw model; Adjusted: adjusted for age, gender, BMI, diabetes, hypertension, smoking status, pre- and postoperative VAS scores, disease duration, occupation, Pfirrmann grade, Modic changes, herniation type, and herniation segment.

For the Paraspinal Muscle Radscore, the AUC was 0.718 (0.627–0.809), with accuracy 0.667, sensitivity 0.692, and specificity 0.672. Internal validation yielded AUCs of 0.701 (leave-one-out), 0.699 (10-fold), and 0.719 (bootstrap). Adjusted improved performance, giving an AUC of 0.822, accuracy 0.759, sensitivity 0.769, and specificity 0.756.

### 3.4 Combined predictive performance assessment of the responsible intervertebral disc and paraspinal muscles

**Figure 3:**
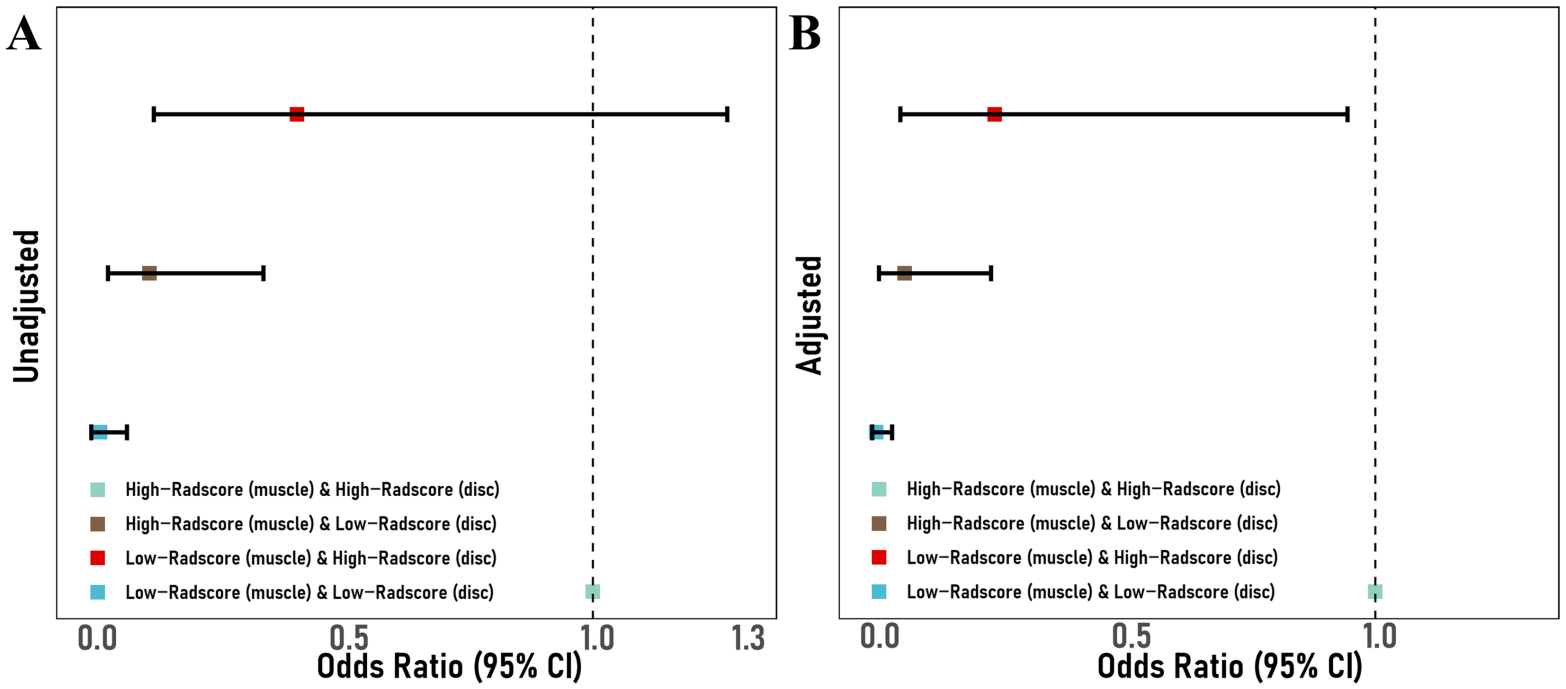
Unadjusted **(A)** and adjusted **(B)** forest plot illustrating the interaction between intervertebral disc Radscore and paraspinal muscle Radscore.

Using the identified cutoffs, patients were classified according to the Intervertebral Disc Radscore (−0.824) and Paraspinal Muscle Radscore (−0.970) into four groups: “High-Radscore (muscle) and High-Radscore (disc),” “Low-Radscore (muscle) and High-Radscore (disc),” “High-Radscore (muscle) and Low-Radscore (disc),” and “Low-Radscore (muscle) and Low-Radscore (disc)” (Figure 3; Table 4). Relative to the High-Radscore (muscle) and High-Radscore (disc) group, all other combinations were protective, with ORs ranging from 0.022 to 0.413 (95% CI 0.005–1.267) and adjusted ORs from 0.009 to 0.245 (95% CI 0.001–0.945).

**Table 4:** Interaction between intervertebral disc and paraspinal muscle.

Variable	*B*	S. E.	*P*	OR	OR 95%CI	
					Lower	Upper
Unadjusted						
High-Radscore (muscle) and High-Radscore (disc)	Ref					
Low-Radscore (muscle) and High-Radscore (disc)	−0.886	0.579	0.126	0.413	0.129	1.267
High-Radscore (muscle) and Low-Radscore (disc)	−2.118	0.561	<0.001	0.120	0.038	0.346
Low-Radscore (muscle) and Low-Radscore (disc)	−3.830	0.698	<0.001	0.022	0.005	0.075
Adjusted						
High-Radscore (muscle) and High-Radscore (disc)	Ref					
Low-Radscore (muscle) and High-Radscore (disc)	−1.408	0.709	0.047	0.245	0.057	0.945
High-Radscore (muscle) and Low-Radscore (disc)	−2.729	0.701	<0.001	0.065	0.015	0.237
Low-Radscore (muscle) and Low-Radscore (disc)	−4.704	0.847	<0.001	0.009	0.001	0.041

Unadjusted: raw model; Adjusted: adjusted for age, gender, BMI, diabetes, hypertension, smoking status, pre- and postoperative VAS scores, disease duration, occupation, Pfirrmann grade, Modic changes, herniation type, and herniation segment.

The combined Intervertebral Disc and Paraspinal Muscle model achieved adjusted and unadjusted AUCs of 0.898 and 0.841, with accuracies of 0.871 and 0.806, sensitivities of 0.821 and 0.744, and specificities of 0.885 and 0.824. Internal validation produced leave-one-out AUCs of 0.811/0.764, 10-fold AUCs of 0.824/0.791, and bootstrap AUCs of 0.784/0.833 (Table 3).

**Figure 4:**
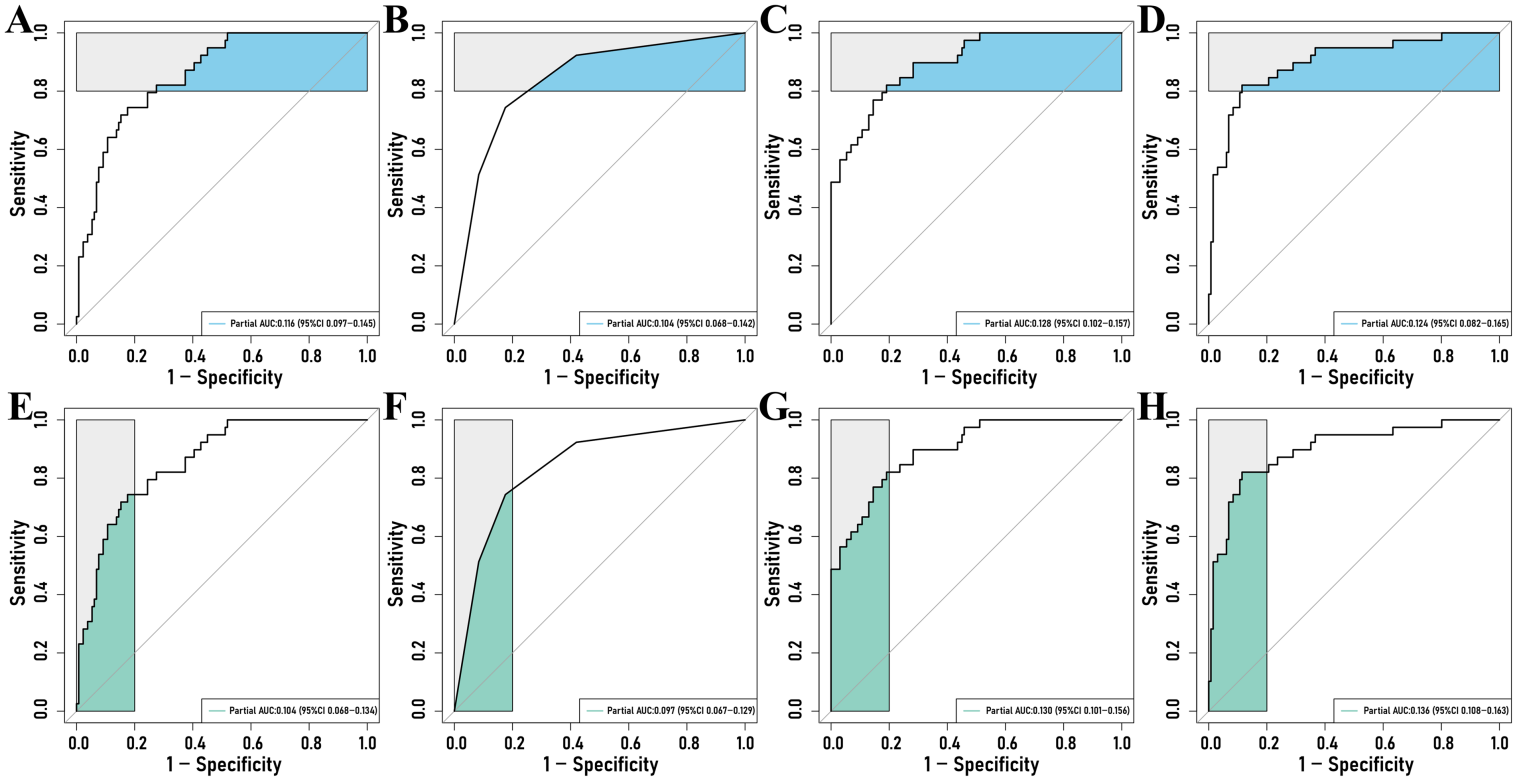
Partial ROC curves for intervertebral disc Radscore and intervertebral disc and paraspinal muscle models at sensitivities of 1.0–0.80 **(A–D)** and specificities of 1.0–0.80 **(E–H)**. **(A,C)** Unadjusted and adjusted partial ROC curves for intervertebral disc Radscore at sensitivities of 1.0–0.80; **(B,D)** Unadjusted and adjusted partial ROC curves for intervertebral disc and paraspinal muscle at sensitivities of 1.0–0.80; **(E,G)** Unadjusted and adjusted partial ROC curves for intervertebral disc Radscore at specificities of 1.0–0.80; **(F,H)** Unadjusted and adjusted partial ROC curves for intervertebral disc and paraspinal muscle at specificities of 1.0–0.80.

**Figure 5:**
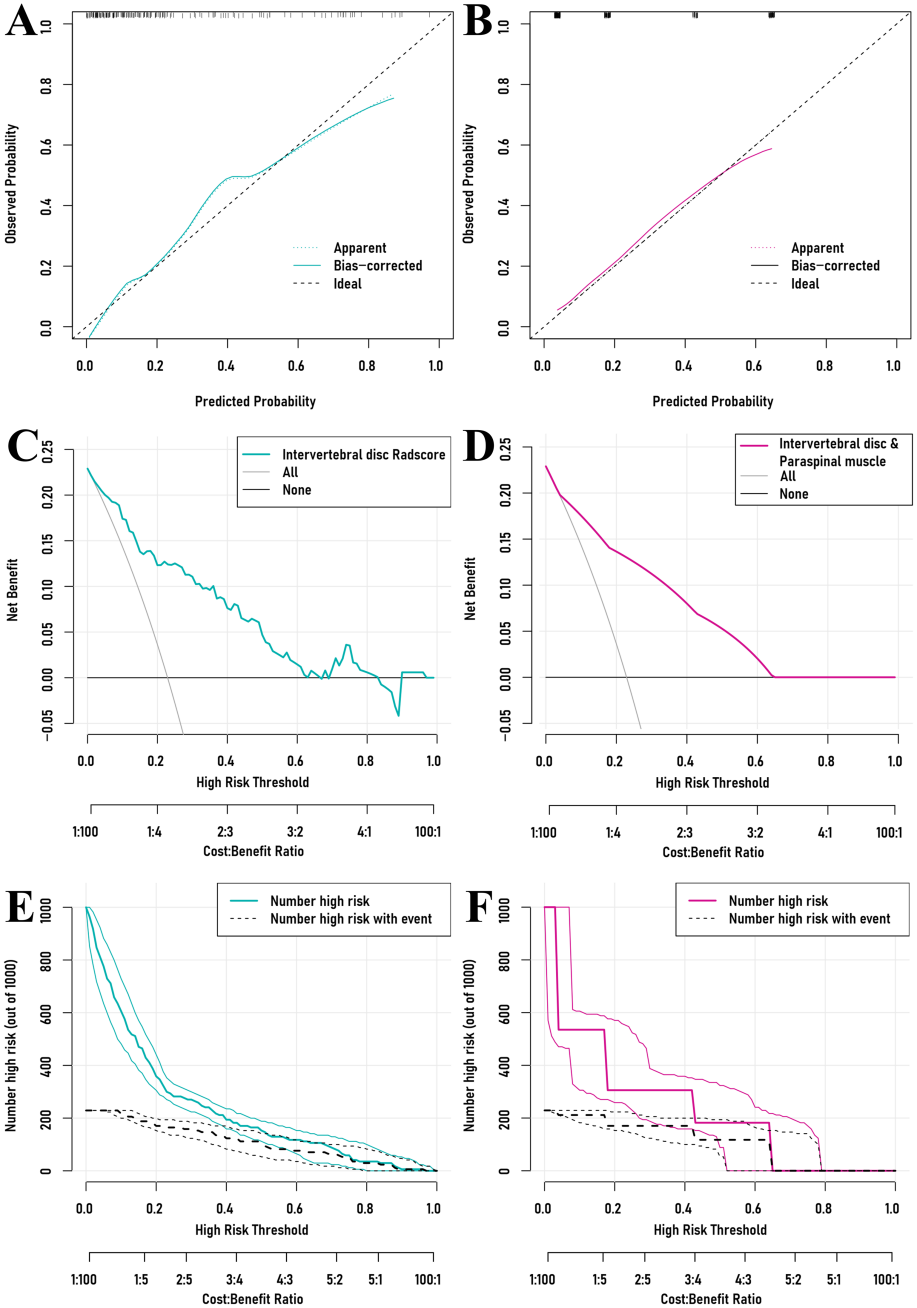
Calibration curves for the intervertebral disc Radscore and the intervertebral disc and paraspinal muscle model **(A,B)**, decision curve analyses **(C,D)**, and clinical impact curves **(E,F)**.

**Figure 6:**
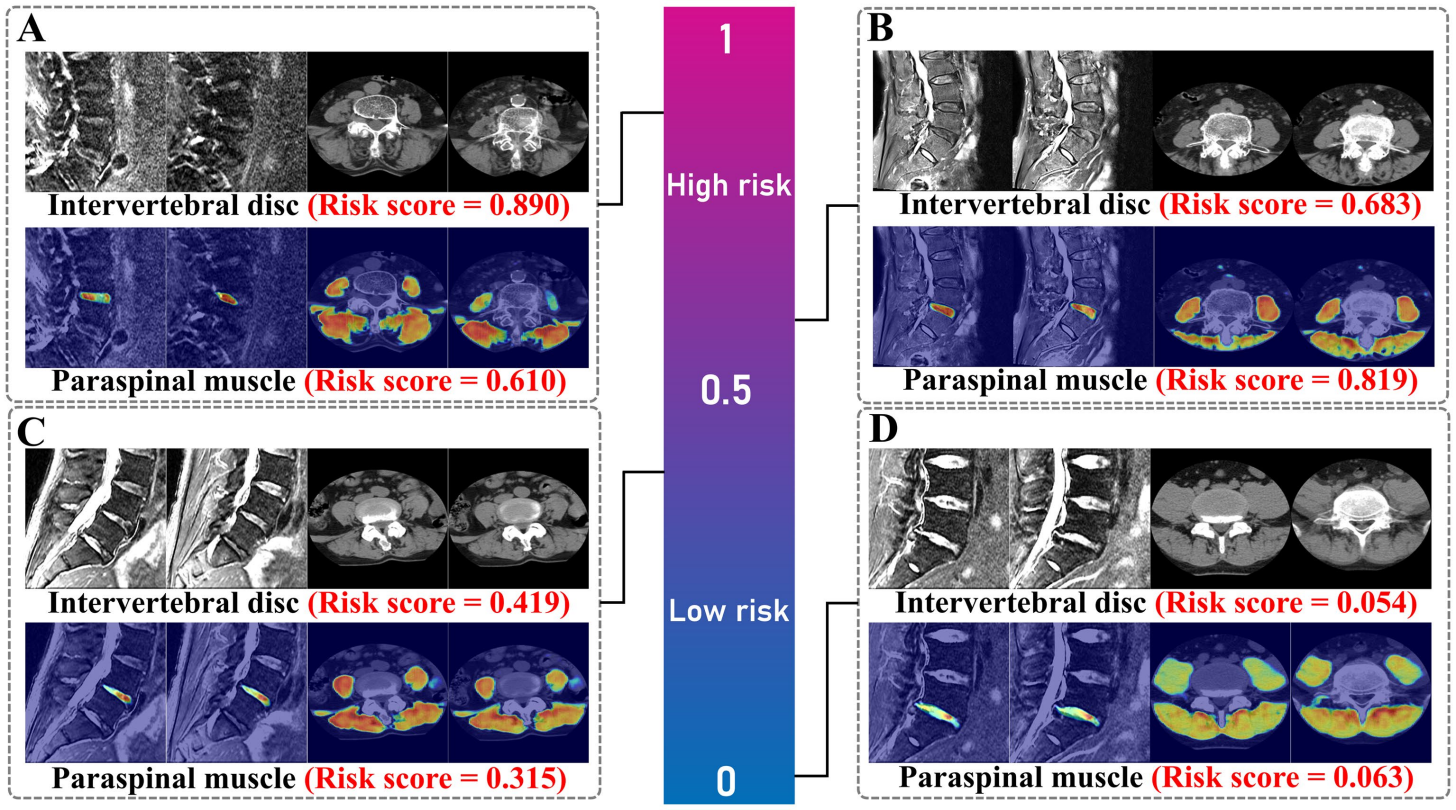
Visualization heatmaps of intervertebral discs and paraspinal muscles across different risk score levels based on UCTransNet. **(A)** high risk, **(B)** medium–high risk, **(C)** medium–low risk, and **(D)** low risk, shown by Grad-CAM and corresponding risk scores.

Partial ROC curves for sensitivity 1–0.80 showed pAUCs of 0.116 for the Intervertebral Disc Radscore and 0.104 for the combined model (*P*_Delong_ = 0.466; adjusted *P*_Delong_ = 0.768). For specificity 1–0.80, pAUCs were 0.104 and 0.097, respectively (*P*_Delong_ = 0.646; adjusted *P*_Delong_ = 0.646) (Figure 4). Both models demonstrated good calibration, and decision curve analysis (DCA) and clinical impact curves (CIC) analyses showed no meaningful differences (Figures 5, 6).

## 4 Discussion

In this study evaluating DL-based radiomics (DL-Radiomics) of the Intervertebral Disc and Paraspinal Muscle for predicting PRLDH, we found that the Intervertebral Disc Radscore demonstrated strong predictive performance (AUC = 0.857, 95% CI: 0.797–0.918). Its performance remained stable even after adjusting for additional factors, including Pfirrmann grade, Modic changes, BMI, and comorbidities, and across different internal validation methods (leave-one-out, 10-fold cross-validation, and bootstrap), with consistently favorable sensitivity and specificity.

Previous studies have suggested that paraspinal muscle characteristics also contribute to PRLDH risk prediction (Tang et al., 2024; Tekin et al., 2025; Kong et al., 2020; Sun et al., 2025; Kocaman et al., 2023); however, these studies typically focused on the L4-L5 segment only. To assess the overall influence of the lower lumbar musculature, we included paraspinal muscles from the L3-S1 segments in our analysis. Although the Paraspinal Muscle Radscore differed significantly between patients with and without PRLDH (*p* < 0.001), its predictive performance (AUC = 0.718, 95% CI: 0.627–0.809) was lower than that reported in prior studies and inferior to the Intervertebral Disc Radscore.

To investigate the combined predictive and risk-stratification potential of the intervertebral disc and paraspinal muscles, we analyzed their interaction. Both adjusted and unadjusted combined models (Intervertebral Disc and Paraspinal Muscle) showed good predictive performance (AUC 0.841–0.898, 95% CI: 0.772–0.958); however, internal validation results were less stable (AUC 0.764–0.833). Importantly, the interaction analysis indicated that, compared with the High-Radscore (muscle) and High-Radscore (disc) group, the Low-Radscore (muscle) and Low-Radscore (disc) combination consistently acted as a protective factor in both unadjusted and adjusted models (*p* < 0.001), supporting its value for postoperative risk stratification.

Finally, comparison of the Intervertebral Disc Radscore with the combined Intervertebral Disc and Paraspinal Muscle model revealed no significant differences in performance for sensitivity and specificity within the 1–0.80 range (*P*_Delong_ = 0.466–0.768). The calibration curve, DCA, and CIC show similar patterns across risk thresholds. The segmentation model achieved robust performance. For the Paraspinal Muscles (Axial view), the model demonstrated excellent accuracy with a Mean DSC of 0.9277 and Mean HD95 of 2.92 mm, reflecting the distinct anatomical boundaries of muscle groups. For the Intervertebral Discs (Sagittal T2WI), the model achieved a Mean DSC of 0.7859 and Mean HD95 of 5.91 mm. While slightly lower than the muscle segmentation metrics, this performance is consistent with the challenges of delineating irregular herniated tissues and complex boundaries in sagittal MRI views. Visual inspection confirmed that the ROIs successfully covered the region of interest for radiomics extraction.

Previous studies investigating the role of the responsible intervertebral disc in PRLDH have primarily focused on the Pfirrmann grading system. Pfirrmann grade reflects pathological changes within the disc, and higher grades correspond to more severe degeneration, which may increase the risk of LDH (Ozden and Silav, 2023). Elevated Pfirrmann grades are associated with reduced water content, loss of proteoglycans, and disruption of collagen fiber architecture within the disc tissue (Wei et al., 2014; Anderson and Tannoury, 2005; Kuzu et al., 2025). These changes compromise the mechanical integrity of the disc, rendering it more susceptible to herniation. For instance, Minin et al. (2025) developed the SpineScan model to enable automated Pfirrmann grading.

In our study, although the Intervertebral Disc Radscore was significantly associated with PRLDH, Pfirrmann grades did not differ between the recurrent and non-recurrent groups (*p* = 0.992), nor between patients with high versus low Intervertebral Disc Radscores (*p* = 0.347). Thus, while prior research has suggested that Pfirrmann grade serves as a primary indicator of disc degeneration and can influence LDH and PRLDH risk, this effect may not always be statistically significant (Bulut et al., 2024). This discrepancy likely arises because Pfirrmann grading relies on T2-weighted signal intensity and morphological features, which capture macroscopic structural changes. In contrast, the DL-based Intervertebral Disc Radscore extracts high-dimensional radiomic features from T2-weighted images that are imperceptible to the human eye, thereby quantifying microstructural heterogeneity within the disc (van der Velden et al., 2022; McSweeney et al., 2025; Fan et al., 2024). Consequently, the Radscore can detect subtle pixel-level pathological and physiological changes within the disc before they manifest as observable morphological differences, allowing earlier and more precise assessment of degeneration and its associated risk (Xie et al., 2023). Even when Pfirrmann grades are identical, underlying disc pathology may vary substantially, and these internal differences likely represent a key determinant of PRLDH occurrence.

The spine, as a multi-joint system, plays a critical role in maintaining posture and facilitating body movement. Panjabi (1992) demonstrated that spinal stability depends on the interplay of three subsystems: the passive subsystem (vertebrae, intervertebral discs, and ligaments), the active subsystem (paraspinal muscles), and the neural control subsystem. These subsystems interact closely, collectively contributing to spinal stability.

Consequently, increasing attention has been given to the role of paraspinal muscles in maintaining spinal stability. Previous studies have shown that degenerative changes in paraspinal muscles, such as fatty infiltration, are associated with PRLDH (Tang et al., 2024; Tekin et al., 2025; Kong et al., 2020; Sun et al., 2025). In our study, we extended the evaluation of paraspinal muscles from the single L4-L5 segment to encompass the full lower lumbar region (L3-S1). Given that the multifidus and erector spinae muscles span multiple vertebral segments, their contribution to spinal stability relies on integrated biomechanical force transmission across the entire muscle chain rather than on isolated segmental effects (Noonan and Brown, 2021). Assessing only the L4-L5 segment may fail to capture the true compensatory capacity of the entire paraspinal muscle group in the lumbar-sacral region, a high-stress area. By including the L3-S1 segments, we aimed to reduce potential selection bias arising from single-segment measurements and provide a more comprehensive evaluation of paraspinal muscle function. Moreover, our findings indicate that paraspinal muscles from L3-S1 exhibit inferior predictive performance for PRLDH compared with the responsible intervertebral disc, suggesting that the primary pathological substrate of PRLDH resides within the disc itself. Although paraspinal muscle degeneration may compromise spinal stability, it appears to act as a secondary or modulatory factor, a relationship further supported by the observed disc-muscle interaction effects.

To explore the interaction between the responsible intervertebral disc and paraspinal muscles in PRLDH development, we analyzed their combined effects. At the macroscopic level, the PRLDH group exhibited a higher proportion of High-Radscore (muscle) and High-Radscore (disc) cases (51.28%), whereas the Non-PRLDH group showed a higher proportion of Low-Radscore (muscle) and Low-Radscore (disc) cases (58.02%), with a significant difference between groups (*p* < 0.001). Notably, a low paraspinal muscle Radscore mitigated the risk associated with a high intervertebral disc Radscore (OR 0.245, 95% CI 0.057–0.945).

Although the combined Intervertebral Disc and Paraspinal Muscle model did not significantly improve predictive performance over the Intervertebral Disc Radscore alone, its value for risk stratification warrants attention. This finding reflects the dynamic compensatory mechanisms between the active and passive subsystems of spinal stability. When the intervertebral disc, as a passive stabilizing structure, exhibits severe degeneration, well-functioning paraspinal muscles can provide critical compensatory protection by enhancing dynamic spinal stiffness and buffering abnormal loads, thereby reducing recurrence risk. For example, Crisco et al. (1992) demonstrated that removing paraspinal muscles from cadaveric lumbar spines resulted in a marked reduction in spinal stability under an average load of 88 N, whereas an intact *in vivo* lumbar spine can withstand an average load of 2,600 N.

However, when both the disc and paraspinal muscles exhibit severe degenerative changes, the spine enters a state of dual structural and functional decompensation. While the combined model offers only limited improvement in overall statistical performance-likely due to the dominant role of disc pathology in PRLDH-it carries important clinical implications. Specifically, even in patients with severely degenerated discs, postoperative rehabilitation aimed at strengthening paraspinal muscle function may leverage muscular compensation to disrupt the vicious cycle of recurrence.

This study has several limitations. First, it is a single-center retrospective study, and the sample size, particularly the number of recurrence events (*n* = 39), represents a primary limitation relative to the high-dimensional radiomics feature space. Although we implemented a strict “coarse-to-fine” feature selection pipeline to reduce the final model to four features (resulting in an Events Per Variable ratio of ~9.75) and verified stability using extensive internal validation (LOOCV and bootstrapping), the risk of overfitting and selection bias cannot be entirely ruled out. The current performance estimates may be optimistic compared to real-world clinical application. Therefore, external validation on larger, multi-center cohorts is essential to confirm the generalizability of our findings before clinical implementation. Second, although we hypothesize that a high Intervertebral Disc Radscore reflects microstructural pathological changes within the disc, direct validation using postoperative histopathological specimens was not performed. Consequently, the precise biological correspondence between radiomic features and specific tissue pathology remains to be elucidated through further basic research. Third, our study primarily focused on local anatomical imaging features and did not fully incorporate global sagittal spinal balance parameters or the biomechanical loading experienced by patients postoperatively, both of which could represent important confounding factors influencing PRLDH risk.

## Data Availability

The original contributions presented in the study are included in the article/Supplementary material, further inquiries can be directed to the corresponding authors.
