# Supplementary material for: Deep learning-radiomics assessment of intervertebral disc and paraspinal muscle heterogeneity for predicting postoperative recurrent lumbar disc herniation
Source: Front Artif Intell. 2026 Feb 4;9:1757269. doi: 10.3389/frai.2026.1757269 (PMC12913562; doi:10.3389/frai.2026.1757269)
Supplement: Supplementary file 1 [file Data_Sheet_1.docx]

Supplementary Material

# Supplementary information

**S1.** **Imaging Biomarker Standardization Initiative (IBSI) reporting structure of the study**

| **Patients** | |
| --- | --- |
| Region of interest | Regions of interest (ROIs) were segmented using 3D Slicer (version 5.8.1; www.slicer.org). The culprit intervertebral disc was delineated on sagittal T2-weighted magnetic resonance imaging (MRI). The bilateral paraspinal muscles (covering L3 to S1 levels) were segmented on lumbar computed tomography (CT) images. The CT modality was selected for muscle segmentation to ensure complete anatomical coverage of the L3-S1 segments, as the field of view (FOV) in routine lumbar MRI was insufficient to cover the entire lower lumbar musculature. |
| Patient Preparation | No specific patient preparation (e.g., fasting) was required. Patients underwent standard lumbar CT and lumbar MRI scans following the institutional protocols. |
| Computed tomography (CT) developing agent | No intravenous or oral contrast agents were administered |
| **Acquisition and Reconstruction** | |
| Protocol | CT Acquisition: Lumbar CT examinations were performed on a [Insert CT Scanner Model, e.g., 64-slice CT scanner] ([Insert Manufacturer, e.g., GE Healthcare, Siemens Healthineers]). The acquisition parameters were fixed as follows: tube voltage 120 kV; tube current 200 mA. The scanning protocol utilized an axial mode (step-and-shoot) focused on the intervertebral disc levels. The scan range covered the L1/2, L2/3, L3/4, L4/5, and L5/S1 intervertebral spaces. For each level, the gantry was angled parallel to the disc space, and 5 slices were acquired per level. Images were reconstructed with a slice thickness of 4 mm.  MRI Acquisition: Magnetic resonance imaging was conducted using a [Insert MRI Scanner Model, e.g., 1.5T/3.0T scanner] ([Insert Manufacturer]). The T2-weighted sequences were acquired with the following parameters: Repetition Time (TR) = 3471.0 ms; Echo Time (TE) = 97.0 ms; slice thickness = 4.0 mm; inter-slice gap = 0.4 mm; Field of View (FOV) = 200 × 200 mm; acquisition matrix = 320 × 320; and Number of Excitations (NEX) = 2. |
| Scanner type | Images were acquired using a 3.0 T MRI scanner (Siemens Healthineers, Erlangen, Germany) and a 64-slice CT scanner (Somatom Definition AS], Siemens Healthineers, Erlangen, Germany) |
| **Delineation** | |
| Software | 3D Slicer (version 5.8.1; www.slicer.org) |
| ROI definition | Regions of interest (ROIs) were defined using a semi-automatic segmentation approach in 3D Slicer (version 5.8.1). The 'Grow from seeds' algorithm was employed for initial ROI generation, followed by slice-by-slice manual refinement using the 'Paint' and 'Erase' tools to ensure accuracy. This process was applied to: (1) the culprit intervertebral disc on sagittal T2-weighted MRI images, strictly delineating the disc boundaries while excluding vertebral endplates; and (2) the bilateral paraspinal muscles on axial CT images at the L3-S1 levels. Muscle segmentation was confined within the fascial boundaries, carefully excluding the vertebral bone, posterior elements, and surrounding subcutaneous fat. |
| Number of experts | To assess inter-observer reproducibility, all images were independently segmented by two interventional radiologists: a junior radiologist (3 years of experience) and a senior radiologist (10 years of experience). |
| Reference image | CT, MRI |
| **Radiomics feature extraction** | |
| Software | Python (version 3.9) |
| Package | PyRadiomics (https://github.com/AIM-Harvard/pyradiomics); PyTorch (https://pytorch.org/get-started/locally) |
| Method | Radiomic features were extracted using PyRadiomics with isotropic resampling (1 mm³), uniform intensity discretization (bin width = 25), and application of multiple image filters including Wavelet, Laplacian of Gaussian (LoG), Square, Square Root, Logarithm, and Exponential transformations. All feature classes (First Order, Shape, GLCM, GLRLM, GLSZM, NGTDM, GLDM) were computed on the original and filtered images. |
| Discretization | Uniform (equal-width binning) |
| Bin width | 25 for CT and MRI |
| Kernels of the filter | Wavelet: coif1 wavelet basis; LoG: sigma values of 1.0, 2.0, 3.0, 4.0, and 5.0 mm; Square, Square Root, Logarithm, Exponential: pixel-wise intensity transformations (no spatial kernel) |
| Biomarker set | First Order Statistics, Shape (3D), Gray Level Co-occurrence Matrix (GLCM), Gray Level Run Length Matrix (GLRLM), Gray Level Size Zone Matrix (GLSZM), Neighboring Gray Tone Difference Matrix (NGTDM), Gray Level Dependence Matrix (GLDM) |
| Exclusion criteria | ICC smaller than 0.75 |

**S2. Formulas for Calculating the Intervertebral Disc Radscore and Paraspinal Muscle Radscore**

Intervertebral disc Radscore = -1.2348 +

0.3238 * *wavelet.HHL_ngtdm_Contrast* +

0.6251 * *DL_445* +

0.4210 * *DL_512* +

0.6661 * *wavelet.LLL_glszm_SizeZoneNonUniformityNormalized* –

0.7324 * *wavelet.HLL_glcm_MCC*

Paraspinal muscle Radscore = 0.1288 +

1.2820 * *wavelet.LHH_glszm_LowGrayLevelZoneEmphasis* +

1.0242 * *DL_191* +

1.1166 * *wavelet.LHL_firstorder_Median* +

0.2984 * *wavelet.HHH_firstorder_Skewness* +

0.2870 * *wavelet.LHH_glrlm_LongRunEmphasis* +

0.9488 * *DL_190* +

0.3058 * *wavelet.HHH_glrlm_GrayLevelNonUniformityNormalized*

**S3.** The segmentation model achieved robust performance. For the Paraspinal Muscles (Axial view), the model demonstrated excellent accuracy with a Mean DSC of 0.9277 and Mean HD95 of 2.92 mm, reflecting the distinct anatomical boundaries of muscle groups. For the Intervertebral Discs (Sagittal T2WI), the model achieved a Mean DSC of 0.7859 and Mean HD95 of 5.91 mm. While slightly lower than the muscle segmentation metrics, this performance is consistent with the challenges of delineating irregular herniated tissues and complex boundaries in sagittal MRI views. Visual inspection confirmed that the ROIs successfully covered the region of interest for radiomics extraction.
